# Radiofrequency Radiation Alters Redox Homeostasis, ATP Status, and Apoptosis-Associated Responses in Human Ovarian Epithelial Cells

**DOI:** 10.3390/cells15191826

**Published:** 2026-10-08

**Authors:** Shaniya Ahmad, Garima Nagar, Neha Yadav, Sanjay Singh, Anju Shrivastava, Rajeev Singh

**Affiliations:** 1 Department of Environmental Science, Faculty of Engineering and Technology, Jamia Millia Islamia, New Delhi 110025, India; ahmadshaniya@gmail.com (S.A.); garimanagar7@gmail.com (G.N.); neha.yadav133@gmail.com (N.Y.); 2 Department of Electronics and Communication Engineering, GLA University, Uttar Pradesh 281406, India; sanjaysanju1001@gmail.com; 3 Department of Zoology, University of Delhi, Delhi 110007, India; ashrivastava@zoology.du.ac.in

**Keywords:** radiofrequency radiation, SKOV3 ovarian carcinoma-derived epithelial cells, oxidative–nitrosative stress, ATP status, apoptosis-associated responses

## Abstract

**Highlights:**

**What are the main findings?**

• 2450 MHz RFR was associated with oxidative–nitrosative stress and a reduction in MTT-based viability/metabolic activity at 48 h, while no significant reduction was detected at 24 h in SKOV3 cells.
• RFR exposure was associated with altered ATP homeostasis without detectable loss of mitochondrial membrane potential (ΔΨm), together with apoptosis-related transcriptional and membrane changes.

**What are the implications of the main findings?**

• These findings define an exposure-associated redox, ATP status, and apoptosis-related cellular stress phenotype under the tested in vitro conditions.
• The findings identify cellular responses associated with 2450 MHz RFR exposure, but the observed ATP changes do not establish mitochondrial respiratory dysfunction or a causal relationship between the measured endpoints.

**Abstract:**

With the expansion of wireless communication technologies, radiofrequency radiation (RFR) has become a persistent component of the environmental exposome. The 2450 MHz frequency is a widely used RF band in wireless and microwave technologies, providing an environmentally relevant model for evaluating non-ionising radiation effects. Although non-ionising, RFR may influence cellular homeostasis through non-thermal stress pathways, yet its cellular effects in ovarian-derived models remain insufficiently defined. This study examined whether 2450 MHz RFR was associated with redox, ATP-related, and apoptosis-associated responses in SKOV3 ovarian epithelial cells. Cells were exposed for 30, 60, and 120 min under controlled conditions at a measured electric field intensity of 13.44 V/m. MTT-based viability/metabolic activity at 24 h and 48 h post-exposure, ROS, nitrite, ATP, mitochondrial membrane potential, apoptosis-related gene expression, and Annexin V-FITC/PI staining were assessed. RFR exposure did not significantly reduce MTT-based viability/metabolic activity at 24 h post-exposure; at 48 h, a significant reduction was observed in the 120-min group compared with the control and 30-min groups. Exposed cells showed progressive ROS accumulation, increased nitrite levels, and changes in ATP levels, with an increase at 30 min followed by lower levels at 60 and 120 min relative to 30 min, while the JC-1 red/green ratio showed no significant change. Apoptosis-associated transcriptional changes were marked by increased Bak1, Nip-2, and Caspase-4 and reduced Bcl-xL and Bag3 expression and a non-significant increase in Bad expression. Annexin V-FITC/PI analysis demonstrated an exposure-duration-associated increase in Annexin V-positive populations, with a predominance of Annexin V^+^/PI^+^ cells following 120 min of exposure. Overall, these findings indicate an exposure-associated cellular stress phenotype involving redox, ATP status, and apoptosis-related alterations in SKOV3 cells under defined in vitro conditions.

## 1. Introduction

The recent development of wireless communication technologies has increased human exposure to anthropogenic electromagnetic fields, making electromagnetic pollution an emerging environmental issue of the digital era [1,2,3]. Although low-intensity non-ionising electromagnetic radiation has always been present in the natural environment, the widespread deployment of modern wireless technologies has markedly increased the level of anthropogenic radiofrequency electromagnetic field (RF-EMF) exposure, establishing RFR as a pervasive environmental factor in contemporary society [4,5,6]. The widespread integration of Internet of Things (IoT)-enabled infrastructure, mobile devices, base stations, smart wearables, Wi-Fi networks, and 5G systems has reshaped the contemporary electromagnetic environment and intensified concerns about the potential biological effects of persistent low-level RFR exposure. However, the biological and health impacts of persistent, low-level RFR exposure remain debated due to inconsistent findings and variability in exposure design, dosimetry, and reproducibility [7,8,9,10,11].

RFR interactions with biological systems involve established thermal mechanisms and less well-understood non-thermal mechanisms. The degree of electromagnetic energy absorption in biological tissues is usually measured in units such as power density and specific absorption rate (SAR) [12,13,14,15,16]. However, in controlled in vitro RFR studies, biological interpretation depends not only on SAR but also on exposure geometry, sample position, field strength, field homogeneity, modulation pattern, antenna-sample distance, culture medium properties, and thermal stability. Therefore, accurate reporting of dosimetric and physical exposure parameters is essential for reproducibility and for comparing biological responses across RFR studies [12,14,17].

Exposure characteristics such as frequency, field strength, and modulation influence RFR propagation and interaction with biological media [18,19,20]. Current exposure guidelines are primarily based on thermal effects, whereas non-thermal interactions remain an active area of investigation for their potential to induce subtle physical and biochemical alterations across cellular, tissue, and organism levels [21,22].

The selected 2450 MHz frequency is relevant to RF energy interaction with biological systems because electromagnetic energy absorption is influenced by frequency-dependent dielectric properties [23,24,25]. Therefore, 2450 MHz was used as a field-characterised in vitro exposure model of a widely encountered RF band. However, in vivo ovarian extrapolation requires caution and SAR-resolved, tissue-specific dosimetry. A growing body of literature suggests that radiofrequency radiation may alter the ovarian structure and function; however, its mechanistic effects on female reproductive biology, particularly human ovarian epithelial cell physiology, remain poorly understood [26,27,28,29,30,31,32]. To better understand the cellular effects of RFR in ovarian-derived systems, it is important to examine responses in human ovarian cellular models [33,34,35]. Reproductive tissues, particularly ovarian cells, appear vulnerable to EMF-induced structural and functional alterations; however, the underlying mechanisms remain poorly defined. Emerging evidence suggests that such perturbations may contribute to ovarian ageing and dysfunction, highlighting the ovary’s susceptibility to environmental stressors [36,37,38].

Accumulating evidence suggests that RFR exposure may perturb cellular redox homeostasis and influence multiple stress-response pathways. Experimental studies have associated RFR exposure with oxidative stress [39,40,41], apoptosis-associated signalling [42,43,44], and mitochondrial dysfunction [39,45,46]. Such molecular alterations may contribute to impaired reproductive function and ovarian cellular dysfunction.

Among the mechanisms implicated in RFR-induced ovarian dysfunction, oxidative stress has emerged as a frequently implicated cellular response. Meta-analytic evidence supports an association between EMF exposure and oxidative stress-related cellular alterations. Elevated ROS may influence mitochondrial function, ATP status, stress-responsive signaling pathways, and apoptosis, positioning mitochondria as both a potential source and target of oxidative stress [47,48]. Mitochondrial dysfunction may further amplify oxidative stress, thereby sustaining cellular injury and compromising ovarian cell integrity and function [42]. Moreover, mitochondrial dysfunction is a key upstream event in the intrinsic apoptotic pathway, promoting Bcl-2 family-regulated loss of mitochondrial integrity, cytochrome c release, and subsequent caspase activation. Consistent with this mechanism, microwave radiation has been reported to alter mitochondrial membrane potential and promote cytochrome c-associated apoptotic signaling in breast-derived cellular models [49,50].

Supporting the involvement of these interconnected pathways, Özgür et al. [51] reported that RF radiation can alter colorectal cancer cell proliferation and inflammatory responses, suggesting that RFR exposure may engage stress-responsive mechanisms linked to redox imbalance, mitochondrial dysfunction, and altered cell-fate signaling.

Similarly, Choi et al. [52] demonstrated that continuous exposure to 1.7 GHz LTE (Long-term evolution) RFR increased intracellular ROS levels, leading to reduced human cell proliferation, G1/S cell-cycle delay, and induction of cellular senescence, rather than apoptosis or DNA damage. Bhartiya et al. [53] further reported that pulsed 3.5 GHz high-power microwave irradiation can induce measurable biological alterations in human cells, including changes in cellular viability, mitochondrial membrane potential, cell-cycle dynamics, and apoptosis-associated pathways. Additional studies have shown that RFR exposure can modulate cellular redox homeostasis, apoptosis, and immune function in a context-dependent manner [39,48].

Nevertheless, the biological effects of RFR exposure remain inconsistent across experimental systems. Romeo et al. [42] highlighted the heterogeneous nature of in vitro evidence in mammalian cells, where radiofrequency exposure has been associated with variable biological outcomes, including induction of apoptosis or minimal effects, largely depending on exposure parameters such as frequency, modulation, and duration. Consistent with this variability, Joushomme et al. [54] reported no detectable effects of environmental radiofrequency radiation exposure on cellular behaviour under either basal or stress-induced conditions, while Haidar et al. [48] provided controlled in vitro evidence that 5G-modulated 3.5 GHz RFR exposure at 0.08 and 4 W/kg does not perturb cytoplasmic or mitochondrial redox homeostasis, elicit oxidative stress adaptation, or compromise Ultraviolet-B radiation (UV-B)-induced DNA repair in human skin cells.

Despite these inconsistencies, the potential reproductive consequences of RFR exposure remain a subject of ongoing concern. Gye and Park et al. [55] reported that RFR exposure may disrupt cellular homeostasis, endocrine regulation, and reproductive function, affecting hormonal balance, gametogenesis, embryonic development, and pregnancy outcomes, thereby indicating potential implications for reproductive health. Collectively, the available evidence suggests that oxidative stress, mitochondrial dysfunction, and altered cell-fate signaling represent plausible cellular responses associated with RFR exposure. However, their coordinated impact in human ovarian epithelial cells remains poorly defined.

Accordingly, the present study investigated the temporal effects of 2450 MHz radiofrequency radiation on SKOV3 ovarian epithelial cells. Using an integrated framework, we evaluated MTT-based viability/metabolic activity at 24 h and 48 h post-exposure, ROS and nitrite accumulation, mitochondrial membrane potential, adenosine triphosphate (ATP) status, regulatory gene expression, and apoptosis-associated membrane changes. Considering that the cellular responses to RFR are highly contingent on exposure design, the observed biological outcomes were interpreted alongside key dosimetric variables, including frequency, modulation pattern, output power, electric field strength, energy-absorption considerations, antenna-sample geometry, thermal stability, polarization, and spatial field homogeneity [12,14,17,20,56]. By examining these interconnected endpoints, we sought to characterize exposure-associated redox, ATP status, and apoptosis-related responses to 2450 MHz in SKOV3 cells and describe cellular responses associated with RFR exposure.

## 2. Materials and Methods

### 2.1. Cell Culture and Maintenance

SKOV3 human ovarian epithelial cancer cells were procured from the National Centre for Cell Science (NCCS), Pune, India. Cells were cultured under aseptic conditions using standard cell- culture procedures. The cells were grown in complete McCoy’s 5A medium (Gibco, Waltham, MA, USA) supplemented with 10% FBS (fetal bovine serum) (FBS; Gibco, Waltham, MA, USA) and 1% penicillin–streptomycin (Gibco, Waltham, MA, USA) solution. Cell cultures were maintained in a humidified incubator (Thermo Fisher Scientific, Waltham, MA, USA) at 37 °C with 5% CO_2_. For routine maintenance, cells were subcultured in complete medium in T75 culture flasks. Once cultures reached 80–90% confluence, they were enzymatically detached using trypsin-phosphate-versene-glucose (TPVG) for further passaging and subsequent experimental assays. Cells in the logarithmic growth phase were used for all experimental assays. Cell number and cell survival were assessed by trypan blue exclusion before seeding/exposure; only cultures with cell survival ≥95% were used for subsequent experimental assays.

### 2.2. Chemicals and Reagents

All chemicals and reagents were of analytical grade and were used according to the manufacturers’ instructions. Cell culture reagents included McCoy’s 5A medium (Gibco, Waltham, MA, USA; Cat. No. 16600082), fetal bovine serum (FBS; Gibco, Waltham, MA, USA; Cat. No. 10270106), penicillin–streptomycin solution (Gibco, Waltham, MA, USA; Cat. No. 15140122), and trypan blue (Gibco, Waltham, MA, USA; Cat. No. 15250061). Trypsin–phosphate versene glucose (TPVG) solution (HiMedia Laboratories, Thane, Maharashtra, India; Cat. No. TCL031), phosphate-buffered saline (PBS; HiMedia Laboratories, Thane, Maharashtra, India; Cat. No. ML023), and bovine serum albumin Fraction V (BSA; HiMedia Laboratories, Thane, Maharashtra, India; Cat. No. MB08353) were used for routine cell culture and experimental procedures. MTT (Cayman Chemical, Ann Arbor, MI, USA; Cat. No. 21795), 2′,7′-dichlorodihydrofluorescein diacetate (DCFH-DA; MedChemExpress, Monmouth Junction, NJ, USA; Cat. No. HYD0940), JC-1 (Cayman Chemical, Ann Arbor, MI, USA; Cat. No. 15003), TriXtract (G-Biosciences, St. Louis, MO, USA; Cat. No. 786652), Ethanol (Empure, Sigma -Aldrich/Merk, 100986, Darmstadt, Germany), cDNA synthesis kit (Bio-Rad Laboratories, Hercules, CA, USA; Cat. No. 1708891), SYBR Green Master Mix (Applied Biosystems, Waltham, MA, USA; Cat. No. A25742), Griess reagent (SRL Chemicals, Mumbai, Maharashtra, India; Cat. No. 35657), ATP assay kit (Invitrogen, Waltham, MA, USA; Cat. No. 3299075), dimethyl sulfoxide (DMSO; Sigma-Aldrich; Cat. No. MB058), Annexin V-FITC/PI Apoptosis Detection Kit (Elabscience, Wuhan, Hubei, China; Cat. No. E-CK-A211), Triton X-100 (Qualigens, Mumbai, Maharashtra, India; Cat. No. Q10655), methanol (Fisher Scientific, Waltham, MA, USA; Cat. No. A452-1), and ethanol (Empure, Qualigens, Mumbai, Maharashtra, India; Cat. No. SP/2322/PB08) were used for the respective experimental assays. Other routine laboratory chemicals were obtained from Sigma-Aldrich/Merck, Qualigens, Fisher Scientific, or HiMedia Laboratories.

### 2.3. RFR Exposure

Radiofrequency radiation exposure and Sham exposures were performed using a previously established exposure approach, as described by Jangid et al. [56] with minor adaptations for SKOV3 ovarian epithelial cells. The 2450 MHz radiofrequency exposure setup consisted of a Keysight N9310A signal generator, CO_2_ incubator, horn antenna, handheld power meter and a Narda 520 field measurement system. Radiofrequency radiation signals of 2450 MHz were generated by a Keysight N9310A signal generator and transmitted through a horn antenna operating in continuous-wave, non-modulated mode. SKOV3 cells in the logarithmic growth phase were exposed to 2450 MHz radiation under controlled incubation at 37 °C and 5% CO_2_. Cell suspensions were cultured in 35-mm-diameter Petri dishes and positioned 2 cm away from the horn antenna. Three dishes were arranged on a flat tray with a centre-to-centre spacing of approximately 36 mm. Since each dish was 35 mm in diameter, the edge-to-edge separation was approximately 1 mm. The dishes were positioned at the centre of the incubator to support homogeneous field distribution at the sample plane. The horn antenna was positioned vertically on the incubator’s upper interior surface and oriented perpendicular to the sample plane. The electric-field intensity was measured at the sample plane before exposure using the Narda 520 system equipped with a near-field probe. The average E-field intensity was 13.44 V/m, and the corresponding peak E-field intensity was 13.82 V/m, with a measurement variation within approximately 2–3%. Sham-exposed cells were placed in the same incubator position for the same exposure durations, with the RF source switched off. Temperature was monitored during exposure and remained comparable between the sham-control and RFR-exposed groups, confirming non-thermal exposure conditions. A sample-specific SAR value was not determined for the present horn-antenna in vitro configuration; therefore, no extrapolated SAR value is reported, and the exposure was characterized using the directly measured E-field, spatial variation, exposure geometry, and thermal stability.

### 2.4. Cellular Studies After RFR Exposure (2450 MHz)

The effect of non-ionizing radiofrequency radiation at 2450 MHz was studied on SKOV3 cells for a period of 30, 60, and 120 min.

#### 2.4.1. MTT-Based Viability/Metabolic Activity Assay

The MTT-based viability/metabolic activity was conducted utilizing the 3-(4,5-Dimethylthiazol-2-yl)-2,5-diphenyltetrazolium bromide (MTT) method. Following irradiation at 2450 MHz, the cells were seeded into a 96-well plate at a density of 1 × 10^5^ cells per well in 100 μL of medium. The cells were then incubated for 24 h and 48 h in a CO_2_ incubator at 37 °C. After the incubation, cells were washed and incubated in 100 μL of medium containing 0.5 mg/mL MTT for 3 h. The cells were washed and solubilized by adding 150 μL of DMSO. After 15 min of incubation at room temperature, the absorbance was measured in an iMark^TM^ Microplate Absorbance Reader (Bio-Rad). % viability was calculated as follows: % Viability = [(Sample Optical Density OD − Blank OD)/(Control OD − Blank OD)] ×100.

#### 2.4.2. Nitrite Estimation

Nitrite concentrations in the cell culture supernatants were measured using the Griess assay. In brief, equal volumes of supernatant were combined with Griess reagent and incubated at room temperature for 10–20 min. Absorbance was subsequently quantified at 490 nm using a microplate reader, with nitrite levels determined relative to a sodium nitrite standard curve.

#### 2.4.3. ROS Determination Using DCFH-DA

To quantify intracellular reactive oxygen species (ROS) levels, SKOV3 cells were exposed to 2450 MHz radiofrequency radiation for defined durations (30, 60, or 120 min) prior to seeding into 96-well plates. Following 24 h incubation at 37 °C in a humidified 5% CO_2_ incubator, the culture medium was aspirated, and cells were gently rinsed with phosphate-buffered saline. Cells were then loaded with 5 μM DCFH-DA probe for 30 min in the dark, after which fluorescence intensity was measured using a Bio-Tek multimode microplate reader.

#### 2.4.4. ATP Quantification Assay

Intracellular ATP concentration measurements were performed using the ATP Determination Kit (Invitrogen) as per the manufacturer’s instructions. Treated cells in brief were broken in ice-cold lysis buffer with the addition of 0.1% Triton X-100. Then, 10 micro liters (µL) of the cell lysate were added to 90 µL of the reaction mixture containing luciferase, d-luciferin, dithiothreitol, and reaction buffer. A Bio-Tek luminometer was used to measure the luminescence produced by the luciferase-catalyzed reaction.

#### 2.4.5. Membrane Potential Assay

JC-1, a lipophilic cationic fluorescent dye, was used to measure mitochondrial membrane potential. Cells were loaded with the dye JC-1 (5 μM) and incubated for 20 min at 37 °C in darkness. The intensities of the red and green fluorescence were then measured using a Bio-Tek multimode microplate reader, and the red-to-green fluorescence ratio indicated the formation of JC-1-dependent J-aggregates. The ratio of red-to-green fluorescence intensities was then used to assess alterations in mitochondrial membrane potential.

#### 2.4.6. Apoptosis Assay

Apoptosis was assessed using the Annexin V-FITC/propidium iodide (PI) apoptosis detection kit according to the manufacturer’s protocol. Immediately after sham or 2450 MHz exposure, both floating and adherent SKOV3 cells were collected, washed twice with cold 1X PBS, and resuspended in 1× binding buffer. Cells were stained with Annexin V-FITC and PI, incubated for 15 min at room temperature in the dark, and analysed immediately by flow cytometry. At least 10,000 events were acquired per sample, and data were analysed using FlowJo software. Debris and doublets were excluded using FSC/SSC and singlet gating. Unstained, Annexin V-FITC-only and PI- only controls were used for compensation and quadrant setting. Annexin V^−^/PI^−^ cells were classified as viable, Annexin V^+^/PI^−^ cells as early apoptotic, Annexin V^+^/PI^+^ cells as late apoptotic/secondary necrotic and Annexin V^−^/PI^+^ cells as necrotic/dead. The Annexin V-positive fraction was calculated as the sum of Annexin V^+^/PI^−^ and Annexin V^+^/PI^+^ cells.

#### 2.4.7. RNA Extraction and Quantitative Real-Time PCR Analysis

After irradiation for 30, 60, and 120 min, 5 × 10^6^ cells were plated in 6-well plates and cultured at 37 °C in a humidified CO_2_ incubator. Total RNA was isolated 24 h later using TriXtract reagent per the manufacturer’s instructions. RNA quantity and quality were assessed via NanoDrop spectrophotometry. cDNA synthesis from 500 ng total RNA utilized the Bio-Rad iScript cDNA Synthesis Kit in a 10 μL reaction volume comprising 2 μL 5X iScript reaction mix, 0.5 μL iScript reverse transcriptase, RNA template, and nuclease-free water. Cycling parameters included priming at 25 °C for 5 min, reverse transcription at 46 °C for 20 min, and inactivation at 95 °C for 1 min. cDNA was diluted 1:30 in nuclease-free water prior to qPCR. Expression of target genes—Caspase-4, Nip2, Bad, Bak1, Bcl-xL, Bag3, and β-Actin- was quantified using the CFX Opus 96 Real-Time PCR System. Table 1 Lists the primers used in the study.

#### 2.4.8. Statistical Analysis

All experiments were independently performed three times (*n* = 3), with measurements conducted in technical triplicates. Technical replicates were averaged before analysis. Data are presented as mean ± SEM and analysed using one-way ANOVA followed by Tukey’s multiple-comparisons test in GraphPad Prism. Statistical significance was defined as * *p* < 0.05, ** *p* < 0.01, *** *p* < 0.001, and **** *p* < 0.0001.

## 3. Results

Electric field (E-field) intensity was measured using a calibrated Narda 520 system. The E-field was 13.44 V/m at 2450 MHz. The measured field parameter remained constant across all exposure durations, with variations falling within the probe’s measurement accuracy.

### 3.1. RFR Exposure Does Not Reduce MTT-Based Viability/Metabolic Activity at 24 h but Reduces It at 48 h Post-Exposure

**Figure 1:**
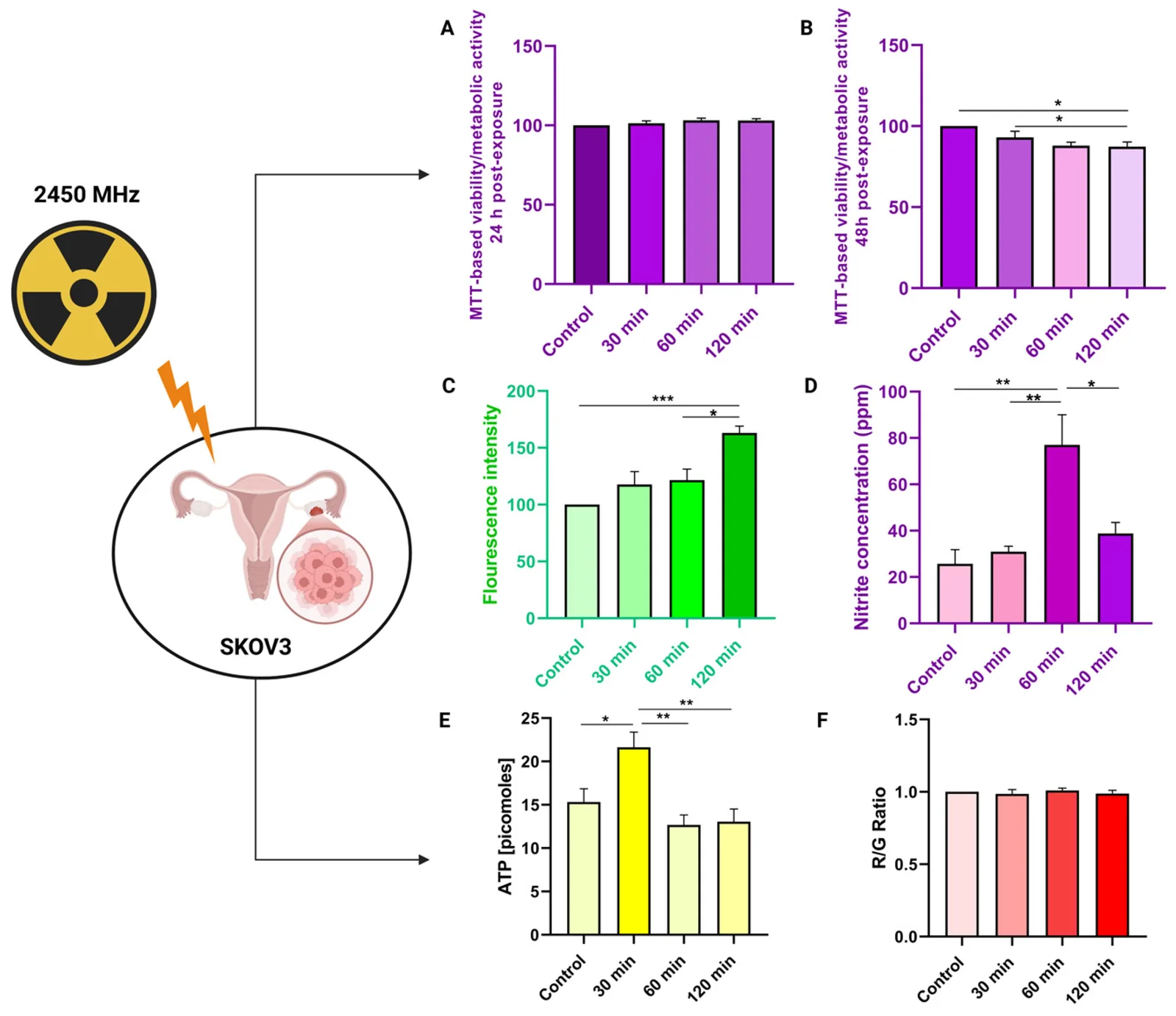
Effect of 2450 MHz RFR exposure on MTT-based viability/metabolic activity, oxidative/nitrosative stress, ATP status, and mitochondrial membrane potential in SKOV3 cells. SKOV3 cells were exposed to 2450 MHz radiofrequency radiation (RFR) for 30, 60, and 120 min under controlled exposure conditions, followed by assessment of MTT-based viability/metabolic activity at 24 and 48 h post-exposure, ROS generation, nitrite accumulation, ATP levels, and mitochondrial membrane potential. (**A**) MTT-based viability/metabolic activity was not significantly altered after 24 h post-RFR exposure, consistent with no significant reduction in MTT-based viability/metabolic activity at 24 h. (**B**) At 48 h post-exposure, MTT-based viability/metabolic activity was significantly lower in the 120-min group than in the control and 30-min groups (* *p* < 0.05); no other pairwise comparisons were significant. (**C**) Intracellular reactive oxygen species (ROS) generation, assessed using DCFH-DA fluorescence intensity, increased following RFR exposure and reached the highest level at 120 min, with significant increases compared with the control and 60 min groups, suggesting enhanced oxidative stress. (**D**) Nitrite concentration increased significantly at 60 min compared with the control and 30 min groups and subsequently decreased significantly at 120 min relative to 60 min, a transient nitrosative stress response. (**E**) Intracellular ATP levels increased significantly at 30 min compared with the control and subsequently decreased significantly at 60 and 120 min relative to the 30 min group, indicating exposure-associated changes in ATP status. (**F**) The JC-1 red/green fluorescence ratio was not significantly altered following RFR exposure, suggesting preservation of mitochondrial membrane potential. Data are presented as mean ± SEM from three independent experiments (*n* = 3). Statistical significance was assessed using one-way ANOVA and Tukey’s multiple-comparisons post hoc test. * *p* < 0.05, ** *p* < 0.01, *** *p* < 0.001, as indicated by the horizontal comparison bars.

The MTT-based viability/metabolic activity assay was performed to assess MTT-based viability/metabolic activity at 24 and 48 h following 2450 MHz RFR exposure for 30, 60, and 120 min (Figure 1A,B). At 24 h post-exposure, MTT-based viability/metabolic activity remained comparable to the control group across all exposure durations, with no significant reduction under the tested conditions (Figure 1A). At 48 h post-exposure, MTT-based viability/metabolic activity showed a modest duration-associated reduction. A significant decrease was observed only in the 120-min group compared with the control and 30-min exposure groups (*p* < 0.05); no other pairwise comparisons were significant (Figure 1B).

### 3.2. Exposure-Associated Oxidative and Nitrosative Stress

SKOV3 cells showed exposure-duration-associated changes in ROS generation and nitrite accumulation following RFR exposure (Figure 1C,D). Fluorometric analysis using DCFH-DA indicated that fluorescence intensity steadily increased with exposure duration. While moderate increases were noted at 30 and 60 min, a profound and statistically significant increase was observed at 120 min (*** *p* < 0.001 vs. control), yielding a 1.6–1.7-fold higher fluorescence intensity than the control group (Figure 1C). Furthermore, fluorescence intensity at 120 min was significantly higher than that at 60 min (* *p* < 0.05). Concurrently, nitrite levels were significantly altered following RFR exposure. Nitrite concentration showed a modest increase at 30 min, and peaked sharply at 60 min, with significant differences observed between control and 60 min (** *p* < 0.01), and between 30 min and 60 min (** *p* < 0.01) (Figure 1D). Although nitrite levels declined at 120 min compared with 60 min, they remained above basal levels, with a significant difference between 60 min and 120 min (* *p* < 0.05).

### 3.3. Altered ATP Homeostasis with Preserved Mitochondrial Membrane Potential

RFR exposure was associated with changes in ATP levels in SKOV3 cells, while JC-1-detected mitochondrial membrane potential was preserved (Figure 1E,F). Intracellular ATP levels showed a non-linear response, with a marked early increase at 30 min as compared to the control group (* *p* < 0.05) (Figure 1E). This early increase was followed by a significant decline at 60 min and 120 min compared with the 30 min exposure group (** *p* < 0.01) (Figure 1E). In contrast, the JC-1 red/green (R/G) fluorescence ratio showed no marked loss of mitochondrial membrane potential (ΔΨm) under the 2450 MHz RFR-exposure conditions (Figure 1F).

### 3.4. Modulation of Apoptotic and Anti-Apoptotic Gene Expression

**Figure 2:**
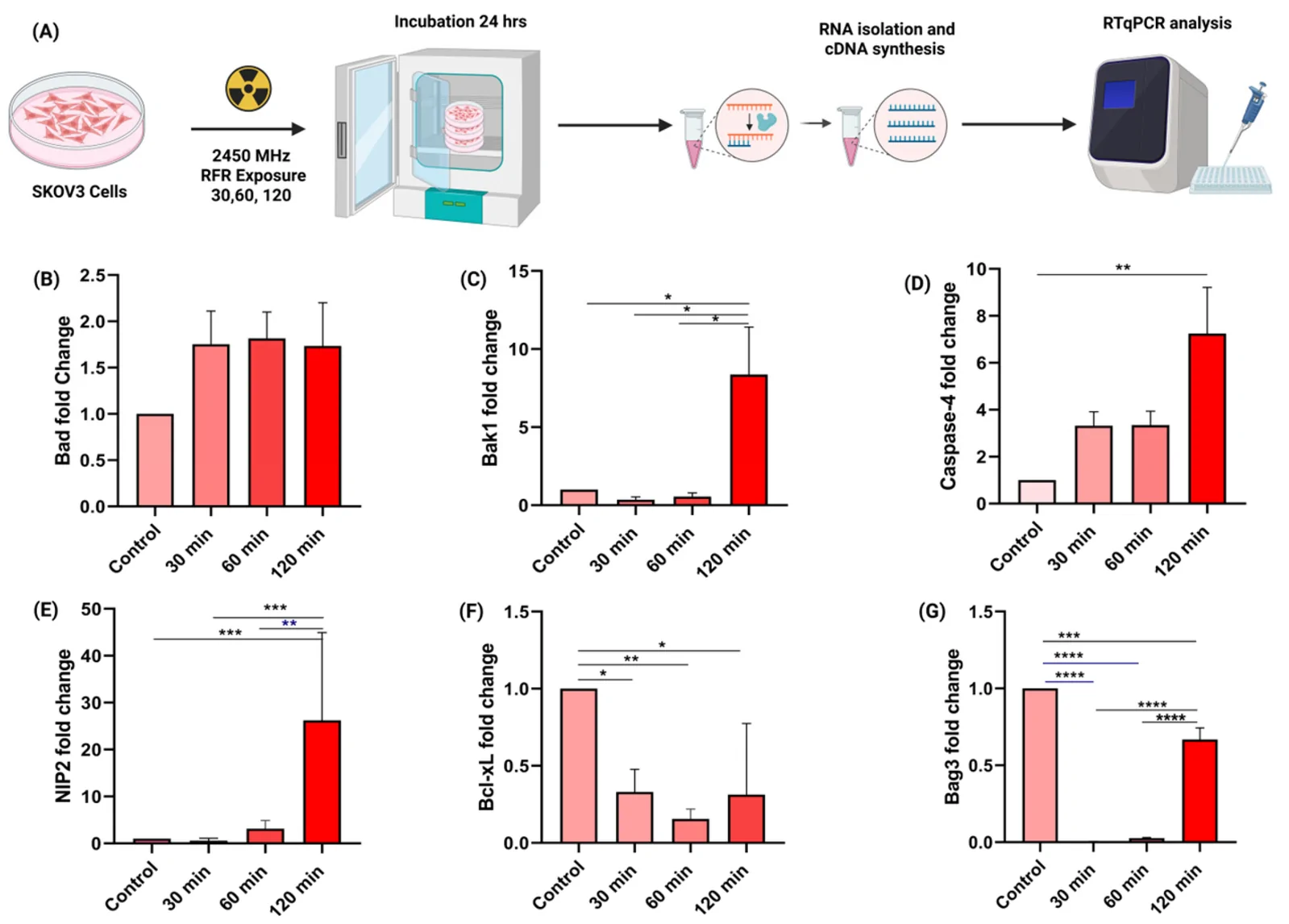
Effect of 2450 MHz RFR exposure on apoptosis-associated gene expression in SKOV3 cells. (**A**) Schematic diagram of the experimental procedure for assessing apoptosis-associated transcriptional responses after exposure to 2450 MHz RFR in SKOV3 cells. Cells were exposed to RFR for 30, 60, or 120 min, followed by 24 h incubation, RNA isolation, cDNA synthesis, and RT-qPCR analysis. (**B**–**G**) Relative mRNA expression analysis of apoptosis-regulatory genes. Bad showed a non-significant increase, whereas Bak1, Caspase-4, and NIP2 were markedly induced, particularly at 120 min. Bcl-xL expression was significantly reduced following RFR exposure, while Bag3 was strongly suppressed at 30 and 60 min with partial recovery at 120 min. Collectively, these changes indicate a shift toward a pro-apoptotic transcriptional profile. Data are presented as mean ± SEM from three independent experiments (*n* = 3). Statistical significance was assessed using one-way ANOVA followed by Tukey’s multiple-comparisons post hoc test. * *p* < 0.05, ** *p* < 0.01, *** *p* < 0.001, **** *p* < 0.0001, as indicated by the horizontal comparison bars.

To elucidate apoptosis-associated transcriptional changes, RT-qPCR was performed for key apoptotic genes and cytoprotective genes at 24 h post-exposure (Figure 2B–G). The pro-apoptotic gene Bad showed an increase in expression at 30 min, which remained elevated at 60 and 120 min; however, these changes were not statistically significant (Figure 2B). Bak1 mRNA expression remained low at 30 and 60 min but increased significantly by approximately 8-fold at 120 min (* *p* < 0.05) (Figure 2C). Caspase-4 expression increased to approximately 3-fold at 30 and 60 min and reached approximately 7-fold at 120 min, with a significant increase at 120 min compared with the control (** *p* < 0.01) (Figure 2D). NIP-2 showed the strongest increase, reaching approximately 26-fold at 120 min, with significant differences between the 120-min group and the control and 30-min groups (*** *p* < 0.001), as well as the 60-min group (** *p* < 0.01) (Figure 2E). Conversely, the anti-apoptotic gene Bcl-xL was significantly downregulated at 30 min (* *p* < 0.05), 60 min (** *p* < 0.01), and 120 min (* *p* < 0.05) (Figure 2F). The expression of Bag3 was markedly suppressed following RFR exposure, with significant reductions at 30 and 60 min (**** *p* < 0.0001) and at 120 min (*** *p* < 0.001) relative to the control (Figure 2G).

### 3.5. FITC-Annexin V Apoptosis Detection

**Figure 3:**
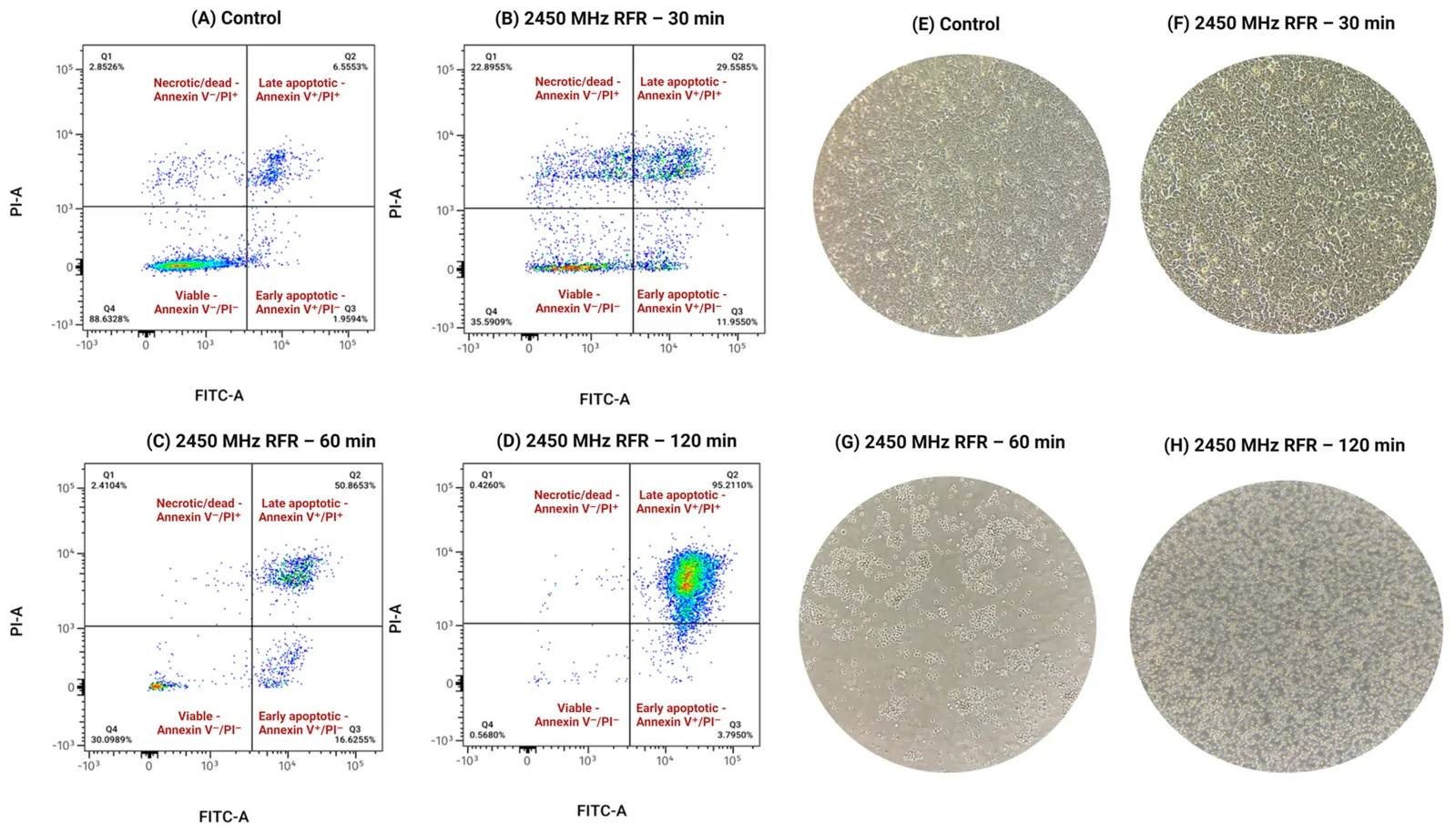
Annexin V/PI staining and cellular morphology associated with 2450 MHz RFR exposure in SKOV3 cells. (**A**–**D**) Representative Annexin V-FITC/PI flow cytometry dot plots of (**A**) control, (**B**) 30 min, (**C**) 60 min, and (**D**) 120 min RFR-exposed cells. Quadrant Q1 represents necrotic/dead cells (Annexin V^−^/PI^+^), Q2 represents late apoptotic/secondary necrotic cells (Annexin V^+^/PI^+^), Q3 represents early apoptotic cells (Annexin V^+^/PI^−^), and Q4 represents viable cells (Annexin V^−^/PI^−^). The *x*-axis represents Annexin V-FITC fluorescence, and the *y*-axis represents PI fluorescence. The Annexin V-positive fraction was calculated as the sum of Annexin V^+^/PI^−^ and Annexin V^+^/PI^+^ populations. Annexin V-positive fractions increased with exposure duration, with a pronounced accumulation in the late apoptotic/secondary necrotic population at 120 min. (**E**–**H**) Representative phase-contrast images of (**E**) control, (**F**) 30 min, (**G**) 60 min, and (**H**) 120 min groups showing exposure-associated alterations in SKOV3 cell morphology.

Annexin V-FITC/PI flow-cytometric analysis showed an exposure-duration-associated increase in Annexin V-positive populations in SKOV3 cells following 2450 MHz RFR exposure (Figure 3A–D). In the control group, most cells remained viable (88.63% Annexin V^−^/PI^−^), while the Annexin V-positive fraction was approximately 8.51%, comprising 1.96% early apoptotic (Annexin V^+^/PI^−^) and 6.56% late apoptotic/secondary necrotic (Annexin V^+^/PI^+^) cells (Figure 3A). After 30 min of RFR exposure, the Annexin V-positive fraction increased to approximately 41.51%, comprising 11.96% early apoptotic and 29.56% late apoptotic/secondary necrotic cells (Figure 3B). At 60 min, the Annexin V-positive fraction increased further to 67.49%, comprising 16.63% early apoptotic and 50.87% late apoptotic/secondary necrotic cells (Figure 3C). Following 120 min of exposure, late apoptotic/secondary necrotic cells constituted the predominant population, accounting for approximately 95.21% of cells, while early apoptotic cells accounted for 3.80% and the viable fraction decreased to 0.56%. The combined Annexin V-positive fraction at 120 min was approximately 99.01% (Figure 3D). Overall, Annexin V/PI analysis showed a marked, exposure-duration-associated increase in Annexin V-positive cells, predominantly within the Annexin V^+^/PI^+^ late apoptotic/secondary necrotic population at 120 min.

## 4. Discussion

The present study found that low-intensity 2450 MHz radiofrequency radiation exposure was associated with exposure-duration-related changes in redox homeostasis, ATP status, MTT-based viability/metabolic activity, and apoptosis-associated responses in SKOV3 human ovarian epithelial cells. Although RFR is generally regarded as non-ionizing and less biologically reactive than ionizing radiation, emerging evidence challenges the assumption that low-level RFR exposure is always biologically inert, particularly in redox-sensitive cellular systems and ovarian tissue-based models [28,47,57].

A salient finding of the present study is the exposure-associated increase in oxidative and nitrosative stress readouts following 2450 MHz RFR exposure. ROS levels increased progressively with exposure duration and reached their highest levels at 120 min, approximately 1.6–1.7-fold above the control (Figure 1C), whereas nitrite accumulation showed an earlier elevation at 30 min, peaking significantly at 60 min, and subsequently declining at 120 min relative to the 60 min group (Figure 1D). Thus, the oxidative and nitrogen-related redox readouts did not follow identical temporal profiles, showing distinct profiles in the oxygen- and nitrogen-related redox readouts. The transient elevation of nitrite, together with progressive ROS accumulation, was associated with an oxidative–nitrosative stress phenotype in SKOV3 cells under the tested exposure conditions. The mechanistic relationship between these distinct temporal profiles was not determined. Similarly, Gulati et al. [58] reported increased lipid oxidation and oxidative DNA lesions in humans living near mobile base stations in association with chronic RFR exposure, although these changes were not statistically significant. ROS and reactive nitrogen species can modify mitochondrial enzymes, membrane lipids, nucleic acids, and redox-sensitive signalling pathways involved in proliferation, stress adaptation, and apoptosis [59,60,61]. NO, in conjunction with superoxide anions, may form peroxynitrite, a reactive nitrogen species implicated in mitochondrial dysfunction, lipid peroxidation, and apoptotic signalling. Thus, the ROS and nitrite readouts are consistent with exposure-associated oxidative–nitrosative imbalance under the tested conditions.

The MTT-based viability/metabolic activity remained comparable to the control at all exposure durations at 24 h post-exposure (Figure 1A).At 48 h post-exposure, MTT-based viability/metabolic activity was significantly reduced only in the 120-min group compared with the control and 30-min groups; no other pairwise comparisons were significant (Figure 1B).This reduction coincided with the maximal increase in DCFH-DA fluorescence (Figure 1C).This temporal concordance supports an association between increased oxidative stress and reduced MTT-based viability/metabolic activity; however, a direct mechanistic relationship cannot be established in the absence of antioxidant intervention or rescue experiments. This interpretation is consistent with studies showing that RFR can increase intracellular ROS in mammalian cell systems [52]. At the same time, the absence of a significant change in 24 h MTT-based viability/metabolic activity indicates that the observed response depended on the interval examined. Because MTT reflects cellular metabolic activity rather than quantifying cell death, the viability findings are most appropriately interpreted as changes in the MTT-based viability/metabolic activity under the conditions used in the present study. Intracellular ATP levels showed a biphasic response, with an early increase at 30 min compared with the control, followed by significant decreases at 60 and 120 min relative to the 30-min group (Figure 1E). The basis of the early ATP increase was not determined. In contrast, the JC-1 red/green fluorescence ratio was not significantly altered following RFR exposure (Figure 1F), indicating no detectable loss of mitochondrial membrane potential (ΔΨm) under the tested conditions. Thus, changes in ATP status occurred without a significant JC-1-detected alteration in mitochondrial membrane potential (ΔΨm), reflecting distinct responses in the measured cellular endpoints without establishing mitochondrial respiratory function [49,50,57].

The apoptosis-associated endpoints also showed exposure-associated transcriptional and membrane changes, with Bad expression showing a non-significant increase (Figure 2B), whereas Bak1 (Figure 2C), Caspase-4 (Figure 2D), and NIP2 (Figure 2E) were significantly induced, particularly following 120 min exposure. Conversely, Bcl-xL was significantly downregulated (Figure 2F), while Bag3 was markedly suppressed following RFR exposure, with the strongest suppression at 30 and 60 min and partial recovery at 120 min (Figure 2G). These transcriptional changes are consistent with a shift toward a pro-apoptotic expression profile but do not demonstrate activation of the corresponding proteins or apoptotic pathways. Bak1 and Bcl-xL are associated with regulation of mitochondrial apoptotic susceptibility, whereas Caspase-4 induction is compatible with potential involvement of stress pathways that may include endoplasmic-reticulum-associated signaling; however, these mechanisms were not functionally established in the present study. In parallel, Annexin V-FITC/PI analysis showed an exposure-duration-associated increase in Annexin V-positive populations, from 8.51% in the control (Figure 3A) to approximately 41.51%, 67.49%, and 99.01% following 30 (Figure 3B), 60 (Figure 3C), and 120 (Figure 3D) min exposure, respectively. At 120 min, approximately 95.21% of cells were Annexin V^+^/PI^+^ and are therefore appropriately classified as late apoptotic/secondary necrotic rather than exclusively apoptotic. Together, the transcriptional and flow-cytometric findings support apoptosis-associated molecular and membrane remodeling following RFR exposure, while further protein-level and functional analyses are required to establish the underlying pathways.

The apparent discrepancy between the immediate Annexin V/PI findings and the 24 h MTT results should be interpreted cautiously because the two assays capture different cellular processes at different assessment intervals. Annexin V/PI analysis was performed immediately after exposure and, following 120 min of RFR exposure, was dominated by an Annexin V^+^/PI^+^ late apoptotic/secondary necrotic population rather than an exclusively early apoptotic population. This time-point-specific membrane-integrity finding does not establish completed, pathway-defined apoptosis. In contrast, MTT analysis at 24 h provides an indirect measure of cellular metabolic activity rather than a direct measurement of cell death (Figure 1A). The unchanged 24 h MTT result neither negates the immediate Annexin V/PI signal nor implies that the assays measure the same process. Recovery during the post-exposure interval, differences in assay sensitivity, and technical contributions related to cell harvesting, membrane stress, or flow-cytometric gating cannot be excluded. Therefore, this pattern should not be interpreted as demonstrating massive immediate cell death followed by unchanged metabolic activity; rather, it indicates assay- and time-point-specific responses that require additional cell-death and recovery measurements before a quantitative cell-death claim can be made.

Contradictory reports also exist in the RFR literature. While several studies have reported oxidative stress, mitochondrial perturbation, altered cell viability/metabolic activity, and apoptosis-associated responses after RF-EMF exposures, other controlled studies have observed minimal or no detectable biological effects. For instance, Choi et al. [52] reported increased intracellular ROS together with reduced proliferation following continuous 1.7 GHz LTE exposure, whereas Joushomme et al. [54] reported no detectable alteration in global cellular behaviour under environmental RF exposure. Similarly, Haidar et al. [48] reported no detectable alteration in cytoplasmic or mitochondrial redox homeostasis, oxidative-stress adaptation, or UV-B-induced DNA repair following controlled 5G-modulated 3.5 GHz RFR exposure in human skin cells. These discrepancies highlight the importance of cell type, exposure duration, frequency, modulation, field strength, SAR, thermal stability, exposure geometry, dosimetry, and endpoint selection when interpreting biological outcomes. Accordingly, the present findings should be viewed as exposure- and model-specific evidence that SKOV3 cells exhibit measurable cellular responses under the field-characterized 2450 MHz conditions used in this study, rather than as evidence of a universal biological response to RFR exposure.

Importantly, ovarian tissue homeostasis is strongly influenced by redox balance, mitochondrial metabolism, and regulated survival signaling [47,57,62]. However, the biological scope of the present model requires careful consideration. SKOV3 is an ovarian epithelial cancer-derived cell line and does not reproduce the physiology or cellular complexity of normal ovarian tissue [33,34]. The present study does not directly assess follicular development, steroidogenesis, oocyte function, fertility, reproductive outcomes and in vivo ovarian injury. Rather, it identifies an exposure-associated cellular stress phenotype in this ovarian carcinoma-derived epithelial cell model. SKOV3 cells do not reproduce the full complexity of normal ovarian tissue; nevertheless, ovarian cancer-derived epithelial cell systems remain useful platforms for mechanistic investigation of ovarian epithelial responses.

The present study found that acute 2450 MHz RFR exposure is associated with coordinated cellular responses in SKOV3 cells under controlled, temperature-stable in vitro conditions. MTT-based viability/metabolic activity remained comparable to the control at all exposure durations at 24 h post-exposure (Figure 1A); however, a significant reduction was observed at 48 h post-exposure, with the greatest reduction occurring following 120 min of RFR exposure (Figure 1B), coinciding with the maximal increase in DCFH-DA fluorescence (Figure 1C). This response was accompanied by transient nitrite elevation and a biphasic ATP response without significant alteration in the JC-1 red/green ratio. In parallel, modulation of apoptosis-associated genes and the exposure-duration-associated increase in Annexin V-positive populations indicate molecular and membrane remodeling consistent with cellular stress. Collectively, these findings characterize an exposure-associated phenotype involving altered redox homeostasis, ATP status, and apoptosis-related responses.

## 5. Conclusions

In conclusion, low-intensity 2450 MHz RFR was associated with measurable redox, metabolic, and apoptosis-associated alterations in SKOV3 cells without a significant reduction in MTT-based viability/metabolic activity at 24 h and with a significant reduction in MTT-based viability/metabolic activity at 48 h. These findings characterize an exposure-associated cellular stress response under defined in vitro conditions but should not be extrapolated directly to normal ovarian function or female fertility. Further validation in normal ovarian models, together with protein-level, mitochondrial functional, and cell-death analyses, will be important to establish the biological significance and underlying mechanisms of these responses. Future studies incorporating oxygen consumption rate (OCR) measurements, respiratory-chain analyses, mPTP assessment, and mitochondrial protein-level measurements will be necessary to further characterize mitochondrial function and its relationship to ATP status.

## Figures and Tables

**Table 1:** Primer sequences were as follows.

Gene	Forward Primer (5′ → 3′)	Reverse Primer (5′ → 3′)
Caspase-4	TACAAGACCCACGTGGAGAA	CTTTGGCCCTTGGAGTTTCA
NIP2	CTGGGATGGCTCAGGAAATG	TGGGGACAAGTTCTGCTAGT
Bad	CCCATCGGAAGTTTTGGGTT	ACATTTGGTAGTGAGCACGG
BAK1	GCCCATTCCCACCATTCTAC	CCTAGTAGGTCCTGCTCCTG
Bcl-xL	ACTCCCAGTCCAAATGTCCT	AGGCTGCTCTTGTAGGAAGT
Bag3	CCCATGACCCATCGAGAAAC	GAGGAGGACAAGGAACTGGA
β-actin	GCAAGCAGGAGTATGACGAG	CACCTTCACCGTTCCAGTTT

## Data Availability

The data supporting the findings of this study are available from the corresponding author upon reasonable request.

## Abbreviations

The following abbreviations are used in this manuscript:

DMSO	Dimethyl sulphoxide
E-field	Electric field
FBS	Fetal bovine serum
IoT	Internet of Things
LTE	Long-Term Evolution
MTT	3-(4,5-Dimethylthiazol-2-yl)-2,5-diphenyltetrazolium bromide
NCCS OCR	National Centre for Cell Science Oxygen Consumption Rate
PBS	Phosphate-buffered saline
PI	Propidium iodide
R/G	Red-to-green fluorescence ratio
RF-EMF	Radiofrequency Electromagnetic field
RFR	Radiofrequency radiation
ROS	Reactive oxygen species
SAR	Specific absorption rate
TPVG	Trypsin–phosphate versene glucose
